# Measuring Parent Satisfaction With Care in Neonatal Intensive Care Units: The EMPATHIC-NICU-USA Questionnaire

**DOI:** 10.3389/fped.2020.541573

**Published:** 2020-10-06

**Authors:** Eileen T. Lake, Jessica G. Smith, Douglas O. Staiger, Kathryn M. Schoenauer, Jeannette A. Rogowski

**Affiliations:** ^1^Center for Health Outcomes and Policy Research, School of Nursing, University of Pennsylvania, Philadelphia, PA, United States; ^2^College of Nursing and Health Innovation, University of Texas at Arlington, Arlington, TX, United States; ^3^Department of Economics, Dartmouth College, Hanover, NH, United States; ^4^Department of Health Policy and Administration, The Pennsylvania State University, University Park, PA, United States

**Keywords:** parent satisfaction, neonatal intensive care unit, nurses, psychometric testing, instrument validation, EMPATHIC, hospitals, instrument adaptation

## Abstract

**Background:** Neonatal intensive care unit (NICU) patient satisfaction is measured as parent satisfaction. Parents are critical to the family-centered care model and can evaluate care. Several EMpowerment of PArents in THe Intensive Care (EMPATHIC) instruments were developed in the Netherlands to measure parent satisfaction with neonatal and pediatric intensive care. EMPATHIC instruments comprise five domains and a total score: information, care and treatment, organization, parental participation, and professional attitude. To our knowledge, the EMPATHIC has not been adapted for USA use.

**Objectives:** (1) To select a relevant EMPATHIC instrument for our study. (2) To expand the content reflecting the role of nurses and the cultural heterogeneity of USA NICU infants. (3) To adapt the selected EMPATHIC instrument to USA English. (4) To establish psychometric properties of the linguistically adapted instrument. (5) To evaluate instrument performance with additional items.

**Methods:** The EMPATHIC-30 was selected based on shortest length, high overlap with neonatal EMPATHIC-N, and availability of a validated Spanish-language version. Six items from the EMPATHIC-N were added, two of which were split into separate items, resulting in the EMPATHIC-38. A neonatal nurse practitioner adapted wording to USA English. Cognitive debriefing was performed with eight NICU parents to evaluate adapted wording. Parent survey data from a study about missed nursing care and NICU parent satisfaction were utilized. Internal consistency of the five domains and overall score was measured by Cronbach's alpha. Spearman's rank correlations were computed for domains and overall score with four validity measures. Differential validity was determined using 13 parent demographic subgroups.

**Results:** Data were from 282 parents. Parent race was predominantly White (61%) or Black (22%). One fifth were Hispanic. The adapted wording was satisfactory. Four of the five EMPATHIC-30 and EMPATHIC-38 domains had Cronbach alphas at or above 0.70, indicating acceptable reliability. Correlations between the domain, total scores, and validity indicators ranged from 0.30 to 0.57, indicating positive, moderate associations. Results were replicated in demographic subgroups. Reliability and validity of the three domains with additional items were better than or equivalent to values for the original.

**Conclusion:** The linguistically adapted EMPATHIC-30-NICU-USA and the expanded EMPATHIC-38-NICU-USA exhibit satisfactory psychometric properties and are suitable for use in USA NICUs.

## Introduction

The parent/infant dyad is the focus of family-centered care (FCC) in the neonatal intensive care unit (NICU) setting. Parent satisfaction influences parents' fulfillment of their parenting roles, which is instrumental to infant growth and development. The purpose of this study was to evaluate the reliability and validity of the USA adaptation of an EMpowerment of PArents in THe Intensive Care (EMPATHIC) instrument. We report the procedure for adaptation as well as the psychometric evaluation.

### Importance of the Family in the Neonatal Intensive Care Unit

A newborn is unique developmentally. Newborns experience rapid growth and development in their first year. During this time, newborns are dependent upon care from the parents. The quality of parenting during this first year is critical to the development of the child. The parents' preparedness to take their child home depends in part on bonding with the infant and the skills they learn from NICU providers to parent an infant who has been critically ill. It is important to understand the family's experiences in the NICU and their satisfaction with it.

### Importance of Parent Satisfaction in the Neonatal Intensive Care Unit

In the evaluation of health care services, the measurement of patient satisfaction has become integral. The Hospital Consumer Assessment of Healthcare Providers and Systems is one of several USA national initiatives focused on patient-centered care (1). Patient satisfaction is considered to reflect the achievement of patient-centered care (2–5). Patient-centered care is defined as providing care that is respectful of and responsive to individual patient preferences, needs, and values and ensuring that patient values guide all clinical decisions (6). Because of the unique dyad of the infant and parent in neonatal care, patient-centered care in this setting entails/requires FCC. Family-centered neonatal care is defined as applying comprehensive and holistic care to neonates and their families with respect (7–9).

In the NICU, evaluating satisfaction with care is essential for health care professionals to provide optimal quality of care considering the family's needs (10). Frequent presence of families at the bedside, in some cases 24 h per day 7 days a week, and the integration of developmental and FCC into practice emphasize the importance of evaluating parent satisfaction in the NICU (10, 11). The importance of parent satisfaction and involvement in the NICU is recognized in the literature (12).

A substantial body of literature exists that demonstrates the effect of FCC and other parental measures on infant outcomes. One study indicated that parental perceptions and attitudes toward their infants' disease can determine the success or failure of treatment plans and neonatal readmission (13). Lv et al. (14) demonstrated that very low birth weight infants might experience improved clinical outcomes, including improved breastfeeding practices and nutritional outcomes, when parents are present and caring for them, and allowing parents to be active partners in care may safeguard infants from complications. Studies have further evaluated the effect of FCC care to improve infant weight gain, increased high-frequency exclusive breast milk feeding at discharge, decreased neonatal readmission, decreased parent stress and anxiety, increased parental adherence to interventions, and increased parental satisfaction (12, 15–18).

### How Is Parent Satisfaction in the Neonatal Intensive Care Unit Evaluated?

Relatively few neonatal parent satisfaction questionnaires have been reported. A systematic review of instruments for assessing parent satisfaction with FCC in NICUs was conducted by Dall'Oglio et al. (19) who found that, from 2006 to 2016, only two validated instruments could properly assess parent satisfaction with FCC in NICUs and be considered as outcome indicators. The validated instruments found in this 2018 systematic review were the EMPATHIC-N developed by Latour et al. (20) and the Neonatal Satisfaction Survey-NSS-13 developed by Hagen et al. (21) The other nine studies evaluated in the review developed or modified previous questionnaires, including the Parent Satisfaction Survey (22), NICU parental satisfaction tool (23), and modified versions of the nurse–parent support tool (24, 25).

### Development of the EMPATHIC Instruments

The 65-item EMPATHIC was developed to measure parent satisfaction in pediatric intensive care units (PICUs) (26). The EMPATHIC was developed in Dutch and published in an English journal (26). The shorter version, the EMPATHIC-30, was derived from the 65-item EMPATHIC (27). There is also a 57-item EMPATHIC-N, which was developed in the Netherlands to measure NICU parent satisfaction (20). The EMPATHIC-N has been translated into Portuguese (28), Greek (29), and Italian (30). The EMPATHIC (26), the EMPATHIC-30 (27), and the EMPATHIC-N (20) all contain five domains of satisfaction: information, care and treatment, organization, parental participation, and professional attitude.

### Psychometric Testing of the EMPATHIC Instruments in Other Languages

The EMPATHIC, EMPATHIC-30, and EMPATHIC-N have been translated into and their psychometric properties have been established in seven languages (20, 26, 28–32). The EMPATHIC domains exhibited Cronbach alphas of 0.73–0.93 in the Netherlands (*n* = 2,046) and from 0.58 to 0.89 in France (*n* = 72) and Switzerland (*n* = 100) (26, 32). The EMPATHIC-30 exhibited domain-level Cronbach alphas of 0.56–0.89 across Spain (*n* = 150) and 0.73–0.81 in the Netherlands (*n* = 3,354) (27, 31). The Cronbach alphas for the total score for the EMPATHIC-30 was 0.93 from the Netherlands (27) and 0.95 from Spain (31) and 0.87 for the EMPATHIC-N from Greece (29). The EMPATHIC-N exhibited domain-level Cronbach alphas above 0.70 in samples from the Netherlands (*n* = 441), Italy (*n* = 162), Greece (*n* = 256), and Brazil (*n* = 40) (20, 28–30).

### Linguistic Adaptation of the EMPATHIC

The EMPATHIC instruments were developed in the Netherlands and tested in Dutch but published in English-speaking journals for dissemination. A research team translated and culturally adapted the EMPATHIC-30 from Dutch to Australian English, resulting in the EMPATHIC-30-AUS, which they validated in the Australian setting of PICU, NICU, and pediatric inpatient wards (33). A USA English adaptation, however, has not been reported. There was, therefore, a need to make sure that the instrument was appropriate for testing among the English-speaking USA population. Beyond instrument translation from one language to another, even within the same language, different variants are needed to account for linguistic and cultural differences. For this reason, the wording of English surveys developed outside the USA need to be adapted to the USA vernacular and cultural context.

When adapting instruments across languages and countries, Flaherty et al. (34) have found that it is imperative that the survey adaptation undergoes an evaluation that involves content, conceptual, semantic, criterion, and technical equivalence to ensure appropriate use in the new location. When translation of survey instruments occurs, it is necessary to translate not only the literal meaning of the words but also the conceptual relation of the word to the context in order to meet the standards of “conceptual equivalence” (35, 36). Failure to meet adaptation requirements of conceptual and contextual equivalence can lead to unreliable and invalid results from a translated instrument (34, 37).

## Materials and Methods

The research team selected and adapted a parent satisfaction instrument in accordance with the COnsensus-based Standards for the selection of health Measurement INstruments (COSMIN) criteria (38). The COSMIN Study Design checklist provides general recommendations on designing studies to evaluate the properties of patient-reported outcome measures (38). Nine other criteria for evaluating specific properties of a patient-reported outcome measurement include: content validity, structural validity, internal consistency, cross-cultural validity/measurement invariance, reliability, measurement error, criterion validity, hypotheses testing for construct validity, and responsiveness (38).

For this paper, we used the general recommendations for study design, which include: having a clear research aim, a clear description of the patient-reported outcome measure (including its development, origin, existing evidence, and context of use), and a clear description of the target population. We also used the content validity guidelines to support our addition of NICU-specific items to the selected instrument.

Consistent with recommendations from Wild et al. (39) regarding linguistic adaptation, we included cognitive debriefing as part of the process to determine the adequacy of linguistic changes. All 10 steps from Wild et al. (39) were not utilized because the EMPATHIC was published previously in English-speaking journals.

Following linguistic adaptation, we evaluated the resulting instrument's properties. Below, we describe instrument selection, expansion, and linguistic adaptation. Lastly, we discuss the approach to evaluating the instrument's psychometric properties.

### Instrument Selection

Of the available EMPATHIC instruments, the EMPATHIC-30 was selected for our NICU study because of its much shorter length than the EMPATHIC-N (30 vs. 57 items), high degree of overlap with the EMPATHIC-N items, and an existing validation for the Spanish language. We were conducting the survey while the infant was in the NICU at the point of discharge and did not want to burden the parents with a much longer instrument at that time. The EMPATHIC-30 contained about half as many items as the EMPATHIC-N, and all but three of the 30 items were identical to EMPATHIC-N items. The three differing items addressed content highly relevant to the NICU parent (being well-prepared for discharge and being asked regularly for our experiences). Furthermore, the significant proportion of Spanish-speaking individuals in the United States calls for inclusion of a Spanish-language version to accommodate these parents. The only validated Spanish version was the EMPATHIC-30.

### Instrument Expansion

For the purpose of our study focusing on nursing care and parent satisfaction in the NICU, we desired items that captured the unique nursing role vis-à-vis parents' developmental needs with their higher-risk newborn. Our intention was to increase the comprehensiveness of the instrument for the NICU setting per the COSMIN criteria. With the developer's permission, the research team, i.e., three nursing faculty, including a neonatal nurse practitioner and two economists experienced in NICU outcomes research, added six items from the 57-item EMPATHIC-N, which was developed for the NICU in order to capture the unique aspects of nursing care of NICU infants and their parents (20). The item selection process entailed reviewing all non-EMPATHIC-30 items of the 57 EMPATHIC-N items as candidates to supplement the EMPATHIC-30, keeping in mind the goal of minimizing survey burden. We selected the six items that most directly related to the nurse's unique role vis-à-vis the parents in the NICU. These items were identified as providing a more complete picture of NICU parent satisfaction. One item addressed emotional support, two items addressed the nurse encouraging and training the parent to care for the child, one item addressed the provider taking action immediately if the child's condition worsened, and one item addressed the team paying sufficient attention to the parents and child in spite of the workload. The last item, “our cultural background was taken into account,” was added to explore the heterogeneous cultural backgrounds of parents in USA NICUs. Two of these items had combined nurses and doctors in the EMPATHIC-N version. We split these into separate items to be more precise about doctors' and nurses' roles. The six added items, with two split into individual nurse and doctor items, yielded a total of eight additional items. We refer to the resulting instrument as the EMPATHIC-38.

### Linguistic Adaptation of the English EMPATHIC-30-NICU-USA and EMPATHIC-38-NICU-USA

The 38 items were evaluated and modified by a USA neonatal nurse practitioner. The adaptation was evaluated by eight NICU parents. A Ph.D. student conducted cognitive debriefing with the parents individually. Based on the results of the debriefing, item wording was revised. The research team members reviewed the adapted items to confirm their wording. For USA Spanish-speaking parents, the Spanish-language version (31) was adapted to the USA cultural and linguistic context, which will be reported elsewhere.

### Evaluating the Reliability and Validity of the English EMPATHIC-30-NICU-USA and EMPATHIC-38-NICU-USA

To evaluate the instrument's psychometric properties, we utilized data from a study of missed nursing care and NICU parent satisfaction (40). These data comprised the EMPATHIC-38-NICU-USA, which has as a subset the EMPATHIC-30-NICU-USA. Thus, both versions were evaluated. By adding the eight derived items from the validated precursor EMPATHIC-N instrument into the same domains of the EMPATHIC-30, the structural validity of each dimension and the scale was upheld. We used Cronbach's alpha to measure the reliability of the five domains and the total score for both the EMPATHIC-38-NICU-USA and the EMPATHIC-30-NICU-USA. We evaluated the correlation of domain score with total scale score. There are four additional items in the EMPATHIC instruments that measure overall parent experience that are not part of calculating the subscales or total score—but are used to assess the construct validity of the domains and total score. These items are described in section *Validity Indicators* below. To assess construct validity, we calculated Spearman's rank correlations between each of the four validity indicators with each domain and total score of the EMPATHIC-30-NICU-USA and EMPATHIC-38-NICU-USA. We evaluated differential validity in subgroups of respondents.

### Recruitment and Data Collection

Secondary data from a study of 30 US NICUs were used. The University of Pennsylvania approved the study protocol from which the secondary data were generated. To serve as a participating site, NICUs obtained institutional review board (IRB) approval through one of three methods: IRB reliance at the University of Pennsylvania, having the project coordinator join the research team as site principal investigator, or obtaining separate IRB approval through their institution. The project coordinator approached eligible parents and explained the study purpose and protocol, including voluntary and confidential participation. The coordinator assured parents that if they chose to not participate, their infant's care and the parents' relationship with the infant's health care providers would not be affected. The coordinator explained to parents that by completing the survey, the parent was giving his or her consent to participate. In each NICU, 10 parents whose infants were scheduled to be discharged in the following 24 h were recruited to answer a survey comprising demographic questions, the EMPATHIC-38, and validity questions in 2018 (40). If an infant died or was transferred to another NICU, the parent was excluded from participating (40). For this psychometric evaluation, only parents who completed the English-language survey version were included. Further information about the NICU sample recruitment approach and inclusion criteria is detailed elsewhere (40).

In participating NICUs, hospital-affiliated project coordinators were identified who were responsible for on-site parent recruitment and overseeing the collection of data (40). At each site, the NICU nurse manager communicated with the project coordinator regarding parents who were eligible to participate (40). Each site's project coordinator recorded the number of eligible parents among those recruited for the purpose of calculating a participation rate (40). When parents were missed or decided not to participate, project coordinators recorded such cases (40). One example of a parent being missed would be if an infant was discharged before the parent could be recruited (40). Prior to data collection, project coordinators attended training webinars about parent recruitment and survey administration guidelines to promote data collection practices that were consistent across NICUs (40). Depending on the hospital's IRB requirements and preferences, a $25 cash or gift card incentive was offered to parent participants (40). IRB approval for this study was obtained from the University of Pennsylvania.

### Measures

#### Parent and Infant Demographics

Demographic questions of the parent include items about race/ethnicity, age, level of completed education, and parent status (i.e., mother, father, both, guardian) of the respondent. Infant characteristic items include whether the infant weighed <1,500 g at birth, in order to capture very low birth weight status, and average length of stay in the NICU in days (Table 1).

**Table 1:** Characteristics of infants and parents.

**Parents**	**Frequency (%)**
**Race/Ethnicity (*****n*** **=** **275)**	
American Indian	3 (1%)
Asian	13 (5%)
Black or African American	61 (22%)
White	169 (61%)
Multiracial	13 (5%)
Other	16 (6%)
**Hispanic or Latino (*****n*** **=** **279)**	
No	223 (80%)
Yes	56 (20%)
**Highest level of education completed (*****n*** **=** **281)**	
Grade school not completed (<8th grade)	1 (0%)
Grade school completed (8th grade completed)	8 (3%)
High school/GED equivalent	118 (42%)
Trade/technical/vocational training	27 (10%)
Associate degree (e.g., AA, AS)	40 (14%)
Bachelor's degree (e.g., BSN)	64 (23%)
Professional degree or doctorate (e.g., Ph.D.)	22 (8%)
Other	1 (0%)
**Age range (*****n*** **=** **281)**	
Under 17 years old	0 (0%)
18–24 years old	68 (24%)
25–34 years old	150 (53%)
35–44 years old	61 (22%)
45–64 years old	2 (1%)
**Who is completing the questionnaire (*****n*** **=** **277)**	
Mother	201 (73%)
Father	39 (14%)
Mother and father together	33 (12%)
Other	4 (1%)
**Infants**	**Frequency (%)**
Infant weighted <1,500 g at birth (*n* = 275)	54 (20%)
	**Mean (*****SD*****)**
Average length of infant stay in NICU, days (*n* = 274)	19.26 (31.05)

#### The EMPATHIC-30 and EMPATHIC-38

The EMPATHIC-30 was developed in the Netherlands to measure the satisfaction of parents of hospitalized children (27). It encompasses the care by nurses and physicians as well as organization and cleanliness. The five domains are listed in tabular results. Items were on a 1-to-6 Likert-type scale, with response options ranging from “certainly no” (1) to “certainly yes” (6), with a “not applicable” option. An overall score is calculated as the mean of all items. An expanded 38-item version was developed for our study as described above. The EMPATHIC-N items referred to “your child,” rather than your “infant” (20), and we retained this wording in our adapted version.

#### Validity Indicators

There were four validity measures: (1) recommend the NICU to others; (2) return to the NICU in the future if needed, both measured on a six-point scale from 0 (certainly no) to 6 (certainly yes); and (3) rate nurses' performance and 4) doctors' performance on a scale from 1 (very bad) to 10 (excellent). The first two validity measures have identical rating scales as the satisfaction items of the measure (six-point scale), while the last two control questions differ in scale (10-point scale) (20).

### Data Analysis

Descriptive statistics were calculated and internal consistency, construct validity, and differential validity were evaluated for the 30- and 38-item versions. Descriptive statistics included means and standard deviations for each item, each domain, and the overall score.

Internal consistency reliability of the five domains and the overall score was measured by Cronbach's alpha, where coefficients of ≥0.7 demonstrate acceptable internal consistency (41). Construct validity was evaluated by computing the correlation between each domain and the overall score of each EMPATHIC version with each of the four validity indicators. Differential validity was evaluated by computing these correlations among demographic subgroups of parent respondents. Significance of correlations was based on a *p* < 0.05. Stata was used for data analyses.

## Results

### Linguistic Adaptation

Eight parents conducted the cognitive debriefing. The parents' ages ranged from teens to 30 s. Six females and two males were included. The parents were of black (*n* = 1) or white (*n* = 5) race and Hispanic ethnicity (*n* = 2). Five parents commented about the survey broadly. They stated that the items made sense to them. Five parents remarked about eight specific items. All of these parents commented about one item, which was originally (UK version): “The team worked hygienically.” The USA adapted version was: “The team was vigilant about maintaining cleanliness.” Based on parent feedback regarding whether the referent was the unit or the infant, the wording was revised to: “The team was attentive about maintaining hygiene.” The other items that the parents commented on were evaluated by the team. In total, minor linguistic adaptations were made to seven items. For example, “The NICU could be reached by telephone” was changed to “The NICU could be reached *easily* by telephone.”

### Parent and Infant Sample Characteristics

Two-hundred eighty-two parents completed the English-language version. Table 1 displays the sample characteristics. Of the 275 parents who reported their race, 61% were White, 22% were Black or African American, 6% were Other, 5% were Multiracial, 5% were Asian, and 1% were Native American. Twenty percent of parents (*n* = 56) reported being Hispanic or Latino. The most common answer for highest level of education completed was “high school or a GED equivalent” (*n* = 118), and 95% of parents had at least a high school education. Three percent of parents indicated grade school completion as their highest level of education. Fifty-three percent of parents were between 25 and 34 years old, 24% were between 18 and 24 years old, 22% were between 35 and 44 years old, and 1% were between 45 and 64 years old. Mothers most often completed this questionnaire alone (73%); however, 14% of fathers completed the questionnaire alone, and 12% of mothers and fathers completed it together. In regard to infant characteristics, 20% of infants for which parents responded about weighed <1,500 g at birth. The average length of stay for infants in the NICU was 19 days.

Across the NICUs, an average of nine parents per NICU responded. In 20 units, all 10 parents completed the English version. From five to 10 parents per unit took the English version. The recruitment approach, which was to recruit the parents of consecutively discharged infants, was designed to achieve a sample representative of the population.

### Item Means and Standard Deviations of EMPATHIC-30-NICU-USA and EMPATHIC-38-NICU-USA

The average percent of missing data was 0.66%. For the item “Our cultural background was taken into account,” 100 respondents marked this item not applicable. Means for the 38 items ranged from 4.93 (“During our child's NICU stay, the staff regularly asked about our experience in the NICU”) to 5.92 (“The nurses prepared us well for our child's discharge”); i.e., near the top of the theoretical range, for which a value of 6 corresponded to “certainly yes.” The most variation (SD) among parent experience ratings was found for the item about staff regularly asking about parent experience in the NICU (*SD* = 1.39). The least variation among parent experience ratings was for the item “The nurses talked to us daily about the care and treatment of our child” (*SD* = 0.35), and the satisfaction for this item was high at a mean of 5.91 out of 6 (Table 2). The items in the EMPATHIC-30-NICU-USA with the highest and lowest means and SDs were the same ones as noted for the EMPATHIC-38-NICU-USA.

**Table 2:** Means and SDs of the statements of EMPATHIC-38-NICU-USA survey.

	***N***	**Mean**	***SD***
**Information**			
The doctors talked to us daily about the care and treatment of our child	282	5.37	1.17
The nurses talked to us daily about the care and treatment of our child	282	5.91	0.35
The doctors clearly informed us about the consequences of our child's treatment	277	5.58	1.01
We received clear information about the examinations and tests	282	5.69	0.70
We received clear information about the effects of the medications	262	5.48	1.10
**Care and treatment**			
The doctors and nurses worked closely together	282	5.69	0.76
The doctors prepared us well for our child's discharge	271	5.60	0.93
The nurses prepared us well for our child's discharge	274	5.92	0.38
The team was attentive to the prevention and treatment of pain in our child	258	5.82	0.58
Our child's comfort was taken into account by the doctor	275	5.78	0.65
Our child's comfort was taken into account by the nurses	278	5.90	0.41
Each day we knew which doctor was responsible for our child's care	281	5.30	1.29
Each day we knew which nurse was responsible for our child's care	281	5.79	0.76
The nurses supported us emotionally^a^	280	5.74	0.71
The doctors supported us emotionally^a^	276	5.24	1.27
The doctors took action immediately when our child's condition worsened^a^	225	5.83	0.61
The nurses took action immediately when our child's condition worsened^a^	229	5.91	0.43
**Organization**			
The NICU team worked efficiently	280	5.86	0.49
The NICU could be reached easily by telephone	260	5.85	0.59
There was enough space around our child's bed	277	5.63	0.93
The NICU was clean	264	5.90	0.36
In the NICU, noise was kept to a minimum	275	5.59	0.95
**Parental participation**			
During our child's NICU stay, the staff regularly asked about our experience in the NICU	276	4.93	1.39
We were actively involved in decision-making about our child's care and treatment	279	5.61	0.80
We were encouraged to stay close to our child	280	5.68	0.80
We had confidence in the doctors	281	5.83	0.51
We had confidence in the nurses	281	5.86	0.43
Even during intensive procedures, we could always stay present with our child	217	5.35	1.34
The nurses encouraged us to help in the care of our child^b^	281	5.89	0.48
The nurses trained us in the specific aspects of our child's care^b^	276	5.90	0.42
**Professional attitude**			
We received sympathy from the doctors	270	5.50	0.99
We received sympathy from the nurses	274	5.81	0.62
The team was attentive about maintaining hygiene	281	5.82	0.62
The team respected the privacy of our child and of us	281	5.88	0.45
The team showed respect for our child and for us	278	5.90	0.37
We felt welcomed at the time of our child's admission to the NICU	278	5.90	0.49
In spite of the workload, sufficient attention was paid to our child and to us by the team^c^	282	5.85	0.50
Our cultural background was taken into account^c^	182	5.49	1.35

a *For the EMPATHIC-38-NICU-USA, four items were added to the care and treatment subscale based on two items from the EMPATHIC-N to determine the extent to which doctors and nurses supported parents emotionally and took action when a change of condition was identified, respectively*.

b *Two items were added from the EMPATHIC-N to the parental participation subscale to account for the role of nurses in encouraging and training parents for the care of their child*.

c *Two EMPATHIC-N items were added to the professional attitude subscale to account for the potential of workload and cultural background to influence parent satisfaction in the USA context*.

### Overall Parent Experience–Validity Indicators

Table 3 presents results for overall parent experience, performance of doctors, and performance of nurses. Parents highly recommended the NICU to others (mean = 5.85, *SD* = 0.56) and would return to the NICU in the future if their child required intensive care (mean = 5.84, *SD* = 0.62). General performance of doctors was highly rated at a mean of 9.41 out of 10 (*SD* = 1.13). For nurses, general performance was similarly high at a mean of 9.62 (*SD* = 0.97).

**Table 3:** Overall parent experience–validity indicators.

**Overall experience**	***N***	**Mean**	***SD***
We would recommend this NICU to anyone whose child required intensive care	277	5.85	0.56
If our child ever required intensive care in the future, we would like to come back to this NICU	273	5.84	0.62
**How would you rate performance in general**	***N***	**Mean**	***SD***
Doctors	281	9.41	1.13
Nurses	278	9.62	0.97

### Comparison of the EMPATHIC-30-NICU-USA and the EMPATHIC-38-NICU-USA

Table 4 presents a comparison of the means, standard deviations, Cronbach's alphas, and number of items for the EMPATHIC-38-NICU-USA and EMPATHIC-30-NICU-USA. For the EMPATHIC-38-NICU-USA, there were three domains in which eight items were added: care and treatment (four items), parental participation (two items), and professional attitude (two items). Reliability results are reported below.

**Table 4:** Descriptive statistics and alpha coefficients for the EMPATHIC-30-USA and−38-USA domains.

	**EMPATHIC-30-NICU-USA**				**EMPATHIC-38-NICU-USA**			
	**Mean**	***SD***	**Alpha**	**Items**	**Mean**	***SD***	**Alpha**	**Items**
Information	5.61	0.65	0.78	5	–	–	–	–
Care and treatment	5.72	0.46	0.75	8	5.69	0.49	0.84	12
Organization	5.76	0.46	0.68	5	–	–	–	–
Parental participation	5.55	0.63	0.81	6	5.64	0.52	0.82	8
Professional attitude	5.80	0.40	0.76	6	5.78	0.41	0.79	8
Total score	5.69	0.42	0.92	30	5.70	0.41	0.94	38

The means and SDs for the *care and treatment* domain and the *professional attitude* domain were essentially equivalent (i.e., <0.1 SD different) across the 30- and 38-item versions.

The *parental participation* domain mean was slightly higher in the EMPATHIC-38-NICU-USA than that in the EMPATHIC-30-NICU-USA (5.64 vs. 5.55). For the *parental participation* domain, the standard deviation was slightly reduced for the EMPATHIC-38 compared to that of the EMPATHIC-30-NICU-USA (*SD* 0.52 vs. 0.63).

### Reliability of EMPATHIC-30-NICU-USA and EMPATHIC-38-NICU-USA

In our NICU parent sample, both the EMPATHIC-38-NICU-USA and the EMPATHIC-30-NICU-USA demonstrated good overall reliability, with overall Cronbach's alphas of 0.9 and 0.92 (Table 4). The Cronbach's alphas for specific domains were very similar across the 38- and 30-item versions, with the exception of the domains with additional items in the 38-item version, which exhibited higher alpha coefficients. Notably, the additional four items in the *care and treatment* domain in the EMPATHIC-38 increased the Cronbach's alpha to 0.84 from 0.75 in the EMPATHIC-30. In both EMPATHIC versions, the Cronbach's alphas for four domains exceeded 0.70: information, *care and treatment, parental participation*, and *professional attitude*. The Cronbach's alpha for the *organization* domain (0.68) was slightly below the standard of 0.70 for measuring scale internal consistency (41).

### Validity of EMPATHIC-30-NICU-USA and EMPATHIC-38-NICU-USA

For the EMPATHIC-30-NICU-USA, the Spearman's rank correlations between each domain and the four validity indicators were small to moderate, positive, and statistically significant. The correlations ranged from 0.30 to 0.57 (Table 5). The highest correlations were for the two performance indicators with the total score ≥0.50. The weakest correlations were for the domains *information* and *organization* (mean *r* of 0.30 and 0.32, respectively, both come back to NICU). The strongest correlations were for the domains *care and treatment* and *professional attitude* (mean *r* of 0.56 and 0.55, respectively, both doctor's performance).

**Table 5:** Spearman's rank correlation of English EMPATHIC-30-NICU-USA and EMPATHIC-38-NICU-USA.

**EMPATHIC-30-NICU-USA**	**Information**	**Care and treatment**	**Organization**	**Parent participation**	**Professional attitude**	**Total score**
Recommend NICU	0.33^*^	0.33^*^	0.38^*^	0.40^*^	0.35^*^	0.40^*^
Come back to NICU	0.30^*^	0.32^*^	0.32^*^	0.40^*^	0.34^*^	0.40^*^
Doctor's performance	0.45^*^	0.56^*^	0.38^*^	0.46^*^	0.55^*^	0.57^*^
Nurse's performance	0.36^*^	0.46^*^	0.40^*^	0.40^*^	0.48^*^	0.50^*^
**EMPATHIC-38-NICU-USA**	**Information**	**Care and treatment**	**Organization**	**Parent participation**	**Professional attitude**	**Total Score**
Recommend NICU	Same as above	0.35^*^	Same as above	0.39^*^	0.38^*^	0.40^*^
Come back to NICU	Same as above	0.34^*^	Same as above	0.40^*^	0.35^*^	0.39^*^
Doctor's performance	Same as above	0.60^*^	Same as above	0.46^*^	0.50^*^	0.57^*^
Nurse's performance	Same as above	0.47^*^	Same as above	0.41^*^	0.49^*^	0.49^*^

* *All correlations are significant p < 0.001*.

For the EMPATHIC-38-NICU-USA, the Spearman's rank correlations between the *information* and *organization* domains and validity items were the same as for the EMPATHIC-30-NICU-USA because items for these domains were not modified. For the *care and treatment* domain, correlations ranged from 0.34 (come back to NICU) to 0.60 (doctor's performance), which were higher than those in the EMPATHIC-30-NICU-USA. For *parent participation* and *professional attitude*, the additional two items in each domain did not change the average correlations with the validity indicators.

For the EMPATHIC-30-NICU-USA, the Pearson's rank correlations between each domain and the overall scale were moderate to large, positive, and statistically significant. The domain correlations to total EMPATHIC-30 score ranged from 0.74 to 0.87 (*p* < 0.001; Table 6). The highest correlation was for the *care and treatment* domain. Similarly, for the EMPATHIC-38-NICU-USA, the Pearson's rank correlations between each domain and the overall scale were moderate to large, positive, and all statistically significant. The highest correlation was between the *care and treatment* domain and the total score (0.91, *p* < 0.001), while the lowest correlation was found with the *organization* domain (0.71, *p* < 0.001). Overall, each domain in both the EMPATHIC instruments was highly correlated with the overall score.

**Table 6:** Pearson's correlation of each domain score with English EMPATHIC-30-NICU-USA and EMPATHIC-38-NICU-USA.

**EMPATHIC-30-NICU-USA**	**Information**	**Care and treatment**	**Organization**	**Parent participation**	**Professional attitude**	**Total score**
Information	1.00	–	–	–	–	–
Care and Treatment	0.65^*^	1.00	–	–	–	–
Organization	0.38^*^	0.57^*^	1.00	–	–	–
Parent Participation	0.51^*^	0.66^*^	0.65^*^	1.00	–	–
Professional Attitude	0.45^*^	0.56^*^	0.48^*^	0.62^*^	1.00	–
Total Score	0.76^*^	0.87^*^	0.74^*^	0.87^*^	0.75^*^	1.00
**EMPATHIC-38-NICU-USA**	**Information**	**Care and treatment**	**Organization**	**Parent participation**	**Professional attitude**	**Total score**
Information	1.00	–	–	–	–	–
Care and Treatment	0.66^*^	1.00	–	–	–	–
Organization	0.38^*^	0.56^*^	1.00	–	–	–
Parent Participation	0.52^*^	0.67^*^	0.64^*^	1.00	–	–
Professional Attitude	0.48^*^	0.68^*^	0.51^*^	0.72^*^	1.00	–
Total Score	0.75^*^	0.91^*^	0.71^*^	0.87^*^	0.83^*^	1.00

* *All correlations are significant p < 0.001*.

Differential validity results are presented in Supplementary Table 1 through M. Differential validity was supported across nine different subgroups by significant correlation coefficients in the hypothesized direction. In five subgroups (i.e., White, non-Hispanic, up to and completion of high school education, above high school education, and mothers as respondents), all correlations were similar in magnitude to the whole sample and were highly significant (*p* < 0.001). In four subgroups (i.e., non-white race, young adults from 18 to 24, over age 24, and infants weighed <1,500 g at birth), some correlations were weaker, although still significant at the *p* < 0.01 or *p* < 0.05 levels.

In four subgroups (i.e., Black, Hispanic, fathers as respondent, and mothers and fathers together as respondent), some correlations between domain scores and differential validity indicators were weaker and non-significant. Notably, the *information* subscale exhibited the weakest correlations with the validity indicators for the black, non-white, Hispanic, and fathers only as respondents subgroups. The young adults had stronger correlation coefficients than the middle-years adults.

For the black subgroup, there was a weak and statistically non-significant correlation between the *information* domain and “recommend NICU,” as well as between the *information* domain and “come back to the NICU.” There was, additionally, a weak and non-statistically significant correlation between the *organization* domain and “nurse's performance” for the black subgroup.

The Hispanic subgroup exhibited weak and non-significant correlations between the *information* domain and “recommend to NICU,” “come back to NICU,” and “nurse's performance,” the *care and treatment* domain and “recommend to NICU” (EMPATHIC-30-NICU-USA only), the *organization* domain and “come back to NICU,” and the *professional attitude* domain and “nurse's performance.” The Hispanic subgroup exhibited a weak and non-significant correlation between the *professional attitude* domain and “come back to NICU” for the EMPATHIC-38-NICU-USA.

The father respondent subgroup exhibited weaker and statistically non-significant correlations between the *information* domain and “recommend NICU,” the *information* domain and “come back to NICU,” and the *care and treatment* domain and “recommend to NICU.” The father respondent subgroup exhibited a weak and significantly non-sufficient correlation between the EMPATHIC-38-NICU-USA *professional attitude* domain and “recommend NICU.”

Compared to father respondents alone, for mother and father respondents, correlations were greater between the *information* domain and “recommend NICU” and between the *information* domain and “come back to NICU” but were statistically insignificant. For mother and father respondents, correlations were weak and statistically non-significant between the *care and treatment* domain and “recommend NICU,” as well as between the *care and treatment* domain and “come back to NICU.” The EMPATHIC-38-NICU-USA total score was not significantly correlated with “come back to the NICU.”

## Discussion

The parent/infant dyad in the NICU is the focal unit of nursing care due to the infant's unique developmental needs. Furthermore, fostering the parent/infant bond and the parent's role in caregiving following discharge are key expectations of care providers and managers during a NICU stay. NICU parent satisfaction is an important indicator of FCC. Among several instruments for measuring NICU parent satisfaction, the EMPATHIC instrument was specific to the critical care setting and includes several domains that address care provider performance. The EMPATHIC-30 was selected for a national NICU study in the USA. To incorporate unique care features of the NICU as compared to pediatric settings, several items were added to the EMPATHIC-30, yielding the EMPATHIC-38. In preparing to survey US NICU parents, the research team identified a compelling need to linguistically and culturally adapt the instrument to be widely available to the culturally diverse racial and ethnic US NICU parents.

This paper describes the linguistic adaptation and the results. We found that both EMPATHIC-NICU-USA versions performed satisfactorily. Although parents were highly satisfied overall, items exhibited variation in their means from a low of 4.93 for “the staff regularly asked about our experience in the NICU” to a high of 5.92 for “the nurses prepared us well for our child's discharge.” There was greater agreement regarding the item “The nurses talked to us daily about the care and treatment of our child,” indicated by a low SD of 0.35. At the other extreme, the largest SD (1.39) was observed for “the staff regularly asked about our experience in the NICU.”

Both EMPATHIC-NICU-USA versions exhibited high reliability for the total score and several domains and good reliability (>0.70) across most domains. One domain, *organization*, had lower-than-desired reliability at 0.68. The domains with additional items in the EMPATHIC-38-NICU-USA had higher reliability (Cronbach's alpha) than the EMPATHIC-30-NICU-USA domains.

The validity of the EMPATHIC-NICU-USA versions was supported by significant correlations with the concurrent validity indicators (nurse/physician performance, recommend NICU, return to NICU if needed). The validity values of the EMPATHIC-NICU-USA demonstrated overall Spearman's rank correlations ranging from 0.30 to 0.60, *p* < 0.001. The domains with additional items and total score in the EMPATHIC-38-NICU-USA had slightly higher (8), equivalent (4), or lower (4) correlation coefficients than the respective EMPATHIC-30-NICU-USA domains. Three of the four weaker correlations were 0.01 lower than the correlation for the comparable domain. One correlation that was 0.05 lower was for the *professional attitude* domain and the physician performance indicator.

The concurrent validity indicators used were consistent with the general satisfaction items used in the countries Spain (31), the Netherlands (20, 26, 27), France (32), Switzerland (32), and Australia (33). The remaining referenced papers did not report Spearman's correlation to demonstrate validity (28, 29). The range of validity values of the EMPATHIC-NICU-USA was consistent with the EMAPTHIC-30 in the Netherlands (0.37–0.51) (20), Australia (0.38–0.69) (33), and France and Switzerland (0.25–0.63) (32). The range of correlation coefficients was slightly lower than those seen by the EMPATHIC-65 in the Netherlands (0.40–0.59) (26) and was slightly higher than the coefficients demonstrated by the EMPATHIC-N in Italy (0.22–0.57) (30).

The EMPATHIC-38-NICU-USA and EMPATHIC-30-NICU-USA demonstrated overall Cronbach's alphas of 0.94 and 0.92. These values are consistent with high Cronbach alphas in the Netherlands (0.93) (27), Spain (0.95) (31), and Australia (0.91) (33) and higher than in Greece (0.87) (29) and Brazil (>0.70 in all domains) (26). The EMPATHIC-NICU-USA versions had similar domain-level alphas (0.68–0.84) as other linguistic adaptations. In both EMPATHIC-NICU-USA versions, the Cronbach's alphas for four domains exceeded 0.70 except for the *organization* domain (0.68). Similarly, in other linguistic adaptations, the *organization* domain occasionally fell below the standard of 0.70. The EMPATHIC-30 exhibited an *organization* domain-level alpha of 0.56 from Spain (*n* = 150) (31) and 0.56 from Australia (*n* = 97) (33), and the EMPATHIC-65 showed an *organization* domain-level alpha of 0.58 from France (*n* = 72) and Switzerland (*n* = 100) (32).

Based on the satisfactory psychometric performance of both versions, we recommend that researchers use the 30-item version for pediatric intensive care settings and the 38-item version for NICU settings.

Our study limitations include substantial “not applicable” responses on one item regarding parents' cultural background being taken into account, which reduced our ability to evaluate its performance. We did not test the EMPATHIC-30 in pediatric intensive care in this study. We encourage USA researchers to test psychometric properties of the linguistically adapted EMPATHIC-30-NICU-USA for PICU settings because the items are identical to the EMPATHIC-30, which was tested originally in the PICU. We did not evaluate whether the structural validity of each domain and the whole scale of the EMPATHIC-30-NICU-USA was upheld in the EMPATHIC-38-NICU-USA after adding items from the EMPATHIC-N, although we added only a few items from the same domains. We note that only three of five domains were altered. The structural validity may show similar factors, which could be tested in future research.

## Conclusion

The linguistically and culturally adapted EMPATHIC-30-NICU-USA and the expanded EMPATHIC-38-NICU-USA exhibited satisfactory psychometric properties and are therefore suitable for use in USA NICUs. These satisfactory properties included high internal consistency reliability (>0.90) for the overall score and good internal consistency reliability (>0.80) for four of the five domains. The *organization* domain was slightly below the acceptable threshold (>0.70). Concurrent validity was supported by significant positive correlations with parent ratings of nurse and physician performance and recommending the NICU to others or willingness to return to the NICU in the future. The 38-item version, which had additional items in three domains (i.e., care and treatment, parent participation, and professional attitude), had higher internal consistency and higher or equivalent correlations for three-quarters of the validity comparisons. The lower correlations were trivially different. We conclude that the EMPATHIC-38-NICU-USA had slightly better performance in measuring parent satisfaction in USA NICUs compared to the EMPATHIC-30-NICU-USA. The validity results were replicated in demographic subgroups. We encourage other researchers to translate this instrument into their languages to increase comparable global evidence and attention to NICU parent satisfaction.

## Data Availability Statement

The datasets generated for this study are available on request to the corresponding author.

## Ethics Statement

The studies involving human participants were reviewed and approved by University of Pennsylvania Institutional Review Board. By completing the survey, participants were giving his or her consent to participate.

## Author Contributions

EL, JS, DS, and JR: study concept, design, and data acquisition. EL, JS, DS, KS, and JR: data analysis, interpretation, and manuscript preparation. All authors reviewed and edited the final manuscript.

## Conflict of Interest

The authors declare that the research was conducted in the absence of any commercial or financial relationships that could be construed as a potential conflict of interest.
