# Supplementary material for: Measuring Parent Satisfaction With Care in Neonatal Intensive Care Units: The EMPATHIC-NICU-USA Questionnaire
Source: Front Pediatr. 2020 Oct 6;8:541573. doi: 10.3389/fped.2020.541573 (PMC7573183; doi:10.3389/fped.2020.541573)
Supplement: Supplementary file 1 [file Data_Sheet_1.docx]

Supplemental Table 1. Spearman’s rank correlation of English EMPATHIC-30-USA and EMPATHIC-38-USA (n=163)

Subsample: White Race

| EMPATHIC-30-USA | Information | Care & Treatment | Organization | Parent Participation | Professional Attitude | Total Score |
| --- | --- | --- | --- | --- | --- | --- |
| Recommend NICU | 0.40*** | 0.33*** | 0.40*** | 0.46*** | 0.40*** | 0.45*** |
| Come back to NICU | 0.33*** | 0.26*** | 0.34*** | 0.42*** | 0.34*** | 0.39*** |
| Doctors Performance | 0.43*** | 0.61*** | 0.46*** | 0.48*** | 0.61*** | 0.59*** |
| Nurses Performance | 0.38*** | 0.46*** | 0.45*** | 0.42*** | 0.46*** | 0.49*** |
| **EMPATHIC-38-USA** | **Information** | **Care & Treatment** | **Organization** | **Parent Participation** | **Professional Attitude** | **Total Score** |
| Recommend NICU | Same as above | 0.38*** | Same as above | 0.45*** | 0.45*** | 0.45*** |
| Come back to NICU | Same as above | 0.31*** | Same as above | 0.41*** | 0.39*** | 0.40*** |
| Doctors Performance | Same as above | 0.65*** | Same as above | 0.48*** | 0.56*** | 0.60*** |
| Nurses Performance | Same as above | 0.46*** | Same as above | 0.42*** | 0.51*** | 0.49*** |

*** correlations are significant p < 0.001

Supplemental Table 2. Spearman’s rank correlation of English EMPATHIC-30-USA and EMPATHIC-38-USA (n=54)

Subsample: Black Race

| EMPATHIC-30-USA | Information | Care & Treatment | Organization | Parent Participation | Professional Attitude | Total Score |
| --- | --- | --- | --- | --- | --- | --- |
| Recommend NICU | 0.10 | 0.40** | 0.49*** | 0.38** | 0.38** | 0.36** |
| Come back to NICU | 0.12 | 0.48*** | 0.44*** | 0.50*** | 0.49*** | 0.44*** |
| Doctors Performance | 0.51*** | 0.44*** | 0.34* | 0.36** | 0.48*** | 0.52*** |
| Nurses Performance | 0.41** | 0.47*** | 0.27 | 0.32* | 0.68*** | 0.54*** |
| **EMPATHIC-38-USA** | **Information** | **Care & Treatment** | **Organization** | **Parent Participation** | **Professional Attitude** | **Total Score** |
| Recommend NICU | Same as above | 0.38** | Same as above | 0.40** | 0.32* | 0.34* |
| Come back to NICU | Same as above | 0.44*** | Same as above | 0.51*** | 0.41** | 0.42** |
| Doctors Performance | Same as above | 0.53*** | Same as above | 0.40** | 0.43** | 0.53*** |
| Nurses Performance | Same as above | 0.48*** | Same as above | 0.34* | 0.58*** | 0.52*** |

*correlations are significant p < 0.05

**correlations are significant p < 0.01

***correlations are significant p < 0.001

Supplemental Table 3. Spearman’s rank correlation of English EMPATHIC-30-USA and EMPATHIC-38-USA (n=105)

Subsample: Non-White Race

| EMPATHIC-30-USA | Information | Care & Treatment | Organization | Parent Participation | Professional Attitude | Total Score |
| --- | --- | --- | --- | --- | --- | --- |
| Recommend NICU | 0.21* | 0.34*** | 0.35*** | 0.28** | 0.27** | 0.33*** |
| Come back to NICU | 0.25* | 0.40*** | 0.32*** | 0.38*** | 0.36*** | 0.40*** |
| Doctors Performance | 0.46*** | 0.47*** | 0.24* | 0.40*** | 0.45*** | 0.53*** |
| Nurses Performance | 0.34*** | 0.46*** | 0.34*** | 0.41*** | 0.52*** | 0.52*** |
| **EMPATHIC-38-USA** | **Information** | **Care & Treatment** | **Organization** | **Parent Participation** | **Professional Attitude** | **Total Score** |
| Recommend NICU | Same as above | 0.31** | Same as above | 0.29** | 0.27** | 0.32** |
| Come back to NICU | Same as above | 0.37*** | Same as above | 0.39*** | 0.30** | 0.39*** |
| Doctors Performance | Same as above | 0.52*** | Same as above | 0.42*** | 0.40*** | 0.54*** |
| Nurses Performance | Same as above | 0.48*** | Same as above | 0.42*** | 0.49*** | 0.52*** |

*correlations are significant p < 0.05

**correlations are significant p < 0.01

***correlations are significant p < 0.001

Supplemental Table 4. Spearman’s rank correlation of English EMPATHIC-30-USA and EMPATHIC-38-USA (n=55)

Subsample: Hispanic

| EMPATHIC-30-USA | Information | Care & Treatment | Organization | Parent Participation | Professional Attitude | Total Score |
| --- | --- | --- | --- | --- | --- | --- |
| Recommend NICU | 0.26 | 0.25 | 0.42** | 0.28* | 0.27* | 0.29* |
| Come back to NICU | 0.22 | 0.31* | 0.22 | 0.38** | 0.31* | 0.36** |
| Doctors Performance | 0.45*** | 0.51*** | 0.44*** | 0.49*** | 0.50*** | 0.57*** |
| Nurses Performance | 0.19 | 0.45*** | 0.44*** | 0.49*** | 0.14 | 0.49*** |
| **EMPATHIC-38-USA** | **Information** | **Care & Treatment** | **Organization** | **Parent Participation** | **Professional Attitude** | **Total Score** |
| Recommend NICU | Same as above | 0.27* | Same as above | 0.28* | 0.37** | 0.29* |
| Come back to NICU | Same as above | 0.29* | Same as above | 0.37** | 0.25 | 0.34* |
| Doctors Performance | Same as above | 0.55*** | Same as above | 0.51*** | 0.46*** | 0.57*** |
| Nurses Performance | Same as above | 0.38** | Same as above | 0.50*** | 0.14 | 0.46*** |

*correlations are significant p < 0.05

**correlations are significant p < 0.01

***correlations are significant p < 0.001

Supplemental Table 5. Spearman’s rank correlation of English EMPATHIC-30-USA and EMPATHIC-38-USA (n=210)

Subsample: Non-Hispanic

| EMPATHIC-30-USA | Information | Care & Treatment | Organization | Parent Participation | Professional Attitude | Total Score |
| --- | --- | --- | --- | --- | --- | --- |
| Recommend NICU | 0.36*** | 0.36*** | 0.40*** | 0.45*** | 0.41*** | 0.42*** |
| Come back to NICU | 0.33*** | 0.35*** | 0.36*** | 0.44*** | 0.39*** | 0.41*** |
| Doctors Performance | 0.46*** | 0.58*** | 0.40*** | 0.48*** | 0.57*** | 0.59*** |
| Nurses Performance | 0.41*** | 0.47*** | 0.40*** | 0.39*** | 0.55*** | 0.51*** |
| **EMPATHIC-38-USA** | **Information** | **Care & Treatment** | **Organization** | **Parent Participation** | **Professional Attitude** | **Total Score** |
| Recommend NICU | Same as above | 0.38*** | Same as above | 0.44*** | 0.44*** | 0.43*** |
| Come back to NICU | Same as above | 0.36*** | Same as above | 0.43*** | 0.42*** | 0.41*** |
| Doctors Performance | Same as above | 0.62*** | Same as above | 0.47*** | 0.51*** | 0.59*** |
| Nurses Performance | Same as above | 0.49*** | Same as above | 0.40*** | 0.57*** | 0.51*** |

*correlations are significant p < 0.05

**correlations are significant p < 0.01

***correlations are significant p < 0.001

Supplemental Table 6. Spearman’s rank correlation of English EMPATHIC-30-USA and EMPATHIC-38-USA (n=118)

Subsample: Up to and Completion of High School Education

| EMPATHIC-30-USA | Information | Care & Treatment | Organization | Parent Participation | Professional Attitude | Total Score |
| --- | --- | --- | --- | --- | --- | --- |
| Recommend NICU | 0.33*** | 0.35*** | 0.40*** | 0.34*** | 0.34*** | 0.39*** |
| Come back to NICU | 0.32*** | 0.40*** | 0.38*** | 0.41*** | 0.35*** | 0.43*** |
| Doctors Performance | 0.46*** | 0.55*** | 0.44*** | 0.51*** | 0.59*** | 0.61*** |
| Nurses Performance | 0.44*** | 0.55*** | 0.46*** | 0.43*** | 0.54*** | 0.58*** |
| **EMPATHIC-38-USA** | **Information** | **Care & Treatment** | **Organization** | **Parent Participation** | **Professional Attitude** | **Total Score** |
| Recommend NICU | Same as above | 0.34*** | Same as above | 0.34*** | 0.38*** | 0.38*** |
| Come back to NICU | Same as above | 0.37*** | Same as above | 0.40*** | 0.37*** | 0.42*** |
| Doctors Performance | Same as above | 0.62*** | Same as above | 0.52*** | 0.52*** | 0.61*** |
| Nurses Performance | Same as above | 0.57*** | Same as above | 0.44*** | 0.59*** | 0.58*** |

*correlations are significant p < 0.05

**correlations are significant p < 0.01

***correlations are significant p < 0.001

Supplemental Table 7. Spearman’s rank correlation of English EMPATHIC-30-USA and EMPATHIC-38-USA (n=150)

Subsample: Above High School Education

| EMPATHIC-30-USA | Information | Care & Treatment | Organization | Parent Participation | Professional Attitude | Total Score |
| --- | --- | --- | --- | --- | --- | --- |
| Recommend NICU | 0.31*** | 0.32*** | 0.38*** | 0.44*** | 0.37*** | 0.41*** |
| Come back to NICU | 0.28*** | 0.27*** | 0.31*** | 0.42*** | 0.36*** | 0.38*** |
| Doctors Performance | 0.44*** | 0.56*** | 0.34*** | 0.42*** | 0.53*** | 0.55*** |
| Nurses Performance | 0.31*** | 0.41*** | 0.39*** | 0.42*** | 0.47*** | 0.45*** |
| **EMPATHIC-38-USA** | **Information** | **Care & Treatment** | **Organization** | **Parent Participation** | **Professional Attitude** | **Total Score** |
| Recommend NICU | Same as above | 0.37*** | Same as above | 0.44*** | 0.39*** | 0.42*** |
| Come back to NICU | Same as above | 0.33*** | Same as above | 0.41*** | 0.36*** | 0.38*** |
| Doctors Performance | Same as above | 0.59*** | Same as above | 0.42*** | 0.48*** | 0.55*** |
| Nurses Performance | Same as above | 0.41*** | Same as above | 0.43*** | 0.44*** | 0.45*** |

*correlations are significant p < 0.05

**correlations are significant p < 0.01

***correlations are significant p < 0.001

Supplemental Table 8. Spearman’s rank correlation of English EMPATHIC-30-USA and EMPATHIC-38-USA (n=63)

Subsample: Young adults 18 to 24

| EMPATHIC-30-USA | Information | Care & Treatment | Organization | Parent Participation | Professional Attitude | Total Score |
| --- | --- | --- | --- | --- | --- | --- |
| Recommend NICU | 0.56*** | 0.40** | 0.43*** | 0.36** | 0.35** | 0.47*** |
| Come back to NICU | 0.52*** | 0.46*** | 0.38** | 0.47*** | 0.35** | 0.53*** |
| Doctors Performance | 0.53*** | 0.55*** | 0.45*** | 0.49*** | 0.50*** | 0.60*** |
| Nurses Performance | 0.43*** | 0.55*** | 0.46*** | 0.54*** | 0.46*** | 0.63*** |
| **EMPATHIC-38-USA** | **Information** | **Care & Treatment** | **Organization** | **Parent Participation** | **Professional Attitude** | **Total Score** |
| Recommend NICU | Same as above | 0.38** | Same as above | 0.37** | 0.43*** | 0.46*** |
| Come back to NICU | Same as above | 0.43*** | Same as above | 0.47*** | 0.42*** | 0.51*** |
| Doctors Performance | Same as above | 0.62*** | Same as above | 0.49*** | 0.43*** | 0.58*** |
| Nurses Performance | Same as above | 0.56*** | Same as above | 0.54*** | 0.47*** | 0.61*** |

*correlations are significant p < 0.05

**correlations are significant p < 0.01

***correlations are significant p < 0.001

Supplemental Table 9. Spearman’s rank correlation of English EMPATHIC-30-USA and EMPATHIC-38-USA (n=204)

Subsample: Over age 24

| EMPATHIC-30-USA | Information | Care & Treatment | Organization | Parent Participation | Professional Attitude | Total Score |
| --- | --- | --- | --- | --- | --- | --- |
| Recommend NICU | 0.25*** | 0.31*** | 0.37*** | 0.41*** | 0.36*** | 0.37*** |
| Come back to NICU | 0.20** | 0.26*** | 0.30*** | 0.38*** | 0.35*** | 0.34*** |
| Doctors Performance | 0.43*** | 0.56*** | 0.37*** | 0.46*** | 0.57*** | 0.57*** |
| Nurses Performance | 0.33*** | 0.43*** | 0.38*** | 0.36*** | 0.49*** | 0.45*** |
| **EMPATHIC-38-USA** | **Information** | **Care & Treatment** | **Organization** | **Parent Participation** | **Professional Attitude** | **Total Score** |
| Recommend NICU | Same as above | 0.34*** | Same as above | 0.40*** | 0.37*** | 0.38*** |
| Come back to NICU | Same as above | 0.31*** | Same as above | 0.38*** | 0.33*** | 0.34*** |
| Doctors Performance | Same as above | 0.59*** | Same as above | 0.46*** | 0.52*** | 0.58*** |
| Nurses Performance | Same as above | 0.44*** | Same as above | 0.37*** | 0.51*** | 0.46*** |

*correlations are significant p < 0.05

**correlations are significant p < 0.01

***correlations are significant p < 0.001

Supplemental Table 10. Spearman’s rank correlation of English EMPATHIC-30-USA and EMPATHIC-38-USA (n=190)

Subsample: Respondents were mothers

| EMPATHIC-30-USA | Information | Care & Treatment | Organization | Parent Participation | Professional Attitude | Total Score |
| --- | --- | --- | --- | --- | --- | --- |
| Recommend NICU | 0.36*** | 0.36*** | 0.41*** | 0.39*** | 0.32*** | 0.42*** |
| Come back to NICU | 0.30*** | 0.33*** | 0.32*** | 0.38*** | 0.28*** | 0.39*** |
| Doctors Performance | 0.45*** | 0.54*** | 0.37*** | 0.44*** | 0.49*** | 0.56*** |
| Nurses Performance | 0.29*** | 0.43*** | 0.36*** | 0.37*** | 0.43*** | 0.46*** |
| **EMPATHIC-38-USA** | **Information** | **Care & Treatment** | **Organization** | **Parent Participation** | **Professional Attitude** | **Total Score** |
| Recommend NICU | Same as above | 0.37*** | Same as above | 0.38*** | 0.39*** | 0.42*** |
| Come back to NICU | Same as above | 0.33*** | Same as above | 0.37*** | 0.33*** | 0.39*** |
| Doctors Performance | Same as above | 0.57*** | Same as above | 0.43*** | 0.43*** | 0.55*** |
| Nurses Performance | Same as above | 0.42*** | Same as above | 0.36*** | 0.44*** | 0.44*** |

*correlations are significant p < 0.05

**correlations are significant p < 0.01

***correlations are significant p < 0.001

Supplemental Table 11. Spearman’s rank correlation of English EMPATHIC-30-USA and EMPATHIC-38-USA (n=39)

Subsample: Respondents were Fathers

| EMPATHIC-30-USA | Information | Care & Treatment | Organization | Parent Participation | Professional Attitude | Total Score |
| --- | --- | --- | --- | --- | --- | --- |
| Recommend NICU | 0.10 | 0.31 | 0.40* | 0.35* | 0.34* | 0.33* |
| Come back to NICU | 0.23 | 0.40** | 0.41** | 0.46** | 0.46** | 0.42** |
| Doctors Performance | 0.45** | 0.52*** | 0.40* | 0.41** | 0.62*** | 0.57*** |
| Nurses Performance | 0.50** | 0.51*** | 0.56*** | 0.44** | 0.62*** | 0.56*** |
| **EMPATHIC-38-USA** | **Information** | **Care & Treatment** | **Organization** | **Parent Participation** | **Professional Attitude** | **Total Score** |
| Recommend NICU | Same as above | 0.30 | Same as above | 0.34* | 0.28 | 0.33* |
| Come back to NICU | Same as above | 0.41** | Same as above | 0.45** | 0.39* | 0.42** |
| Doctors Performance | Same as above | 0.58*** | Same as above | 0.45** | 0.47** | 0.58*** |
| Nurses Performance | Same as above | 0.54*** | Same as above | 0.52*** | 0.56*** | 0.57*** |

*correlations are significant p < 0.05

**correlations are significant p < 0.01

***correlations are significant p < 0.001

Supplemental Table 12. Spearman’s rank correlation of English EMPATHIC-30-USA and EMPATHIC-38-USA (n=31)

Subsample: Respondents were Both Fathers and Mothers

| EMPATHIC-30-USA | Information | Care & Treatment | Organization | Parent Participation | Professional Attitude | Total Score |
| --- | --- | --- | --- | --- | --- | --- |
| Recommend NICU | 0.34 | 0.16 | 0.25 | 0.48** | 0.46** | 0.38* |
| Come back to NICU | 0.31 | 0.16 | 0.24 | 0.47** | 0.45* | 0.36* |
| Doctors Performance | 0.50** | 0.69*** | 0.41* | 0.58*** | 0.77*** | 0.68*** |
| Nurses Performance | 0.55** | 0.52** | 0.45* | 0.51** | 0.55** | 0.64*** |
| **EMPATHIC-38-USA** | **Information** | **Care & Treatment** | **Organization** | **Parent Participation** | **Professional Attitude** | **Total Score** |
| Recommend NICU | Same as above | 0.28 | Same as above | 0.48** | 0.44* | 0.36* |
| Come back to NICU | Same as above | 0.26 | Same as above | 0.47** | 0.43* | 0.34 |
| Doctors Performance | Same as above | 0.79*** | Same as above | 0.58*** | 0.83*** | 0.72*** |
| Nurses Performance | Same as above | 0.61*** | Same as above | 0.51** | 0.66*** | 0.66*** |

*correlations are significant p < 0.05

**correlations are significant p < 0.01

***correlations are significant p < 0.001

Supplemental Table 13. Spearman’s rank correlation of English EMPATHIC-30-USA and EMPATHIC-38-USA (n=51)

Subsample: Infants weighed < 1,500 grams at birth

| EMPATHIC-30-USA | Information | Care & Treatment | Organization | Parent Participation | Professional Attitude | Total Score |
| --- | --- | --- | --- | --- | --- | --- |
| Recommend NICU | 0.41** | 0.42** | 0.57*** | 0.47*** | 0.41** | 0.50*** |
| Come back to NICU | 0.40** | 0.42** | 0.45*** | 0.48*** | 0.46*** | 0.50*** |
| Doctors Performance | 0.38** | 0.52*** | 0.37** | 0.46*** | 0.67*** | 0.55*** |
| Nurses Performance | 0.43** | 0.53*** | 0.55*** | 0.57*** | 0.48*** | 0.61*** |
| **EMPATHIC-38-USA** | **Information** | **Care & Treatment** | **Organization** | **Parent Participation** | **Professional Attitude** | **Total Score** |
| Recommend NICU | Same as above | 0.38** | Same as above | 0.47*** | 0.52*** | 0.47*** |
| Come back to NICU | Same as above | 0.41** | Same as above | 0.49*** | 0.52*** | 0.48*** |
| Doctors Performance | Same as above | 0.61*** | Same as above | 0.48*** | 0.55*** | 0.58*** |
| Nurses Performance | Same as above | 0.57*** | Same as above | 0.57*** | 0.55*** | 0.61*** |

*correlations are significant p < 0.05

**correlations are significant p < 0.01

***correlations are significant p < 0.001
